# Identifying the Role of Common Interests in Online User Trust Formation

**DOI:** 10.1371/journal.pone.0121105

**Published:** 2015-07-10

**Authors:** Lei Ji, Jian-Guo Liu, Lei Hou, Qiang Guo

**Affiliations:** Research Center of Complex Systems Science, University of Shanghai for Science and Technology, Shanghai, People’s Republic of China; Hangzhou Normal University, CHINA

## Abstract

Despite enormous recent efforts in detecting the mechanism of the social relation formation in online social systems, the underlying rules between the common interests and social relations are still under dispute. Do online users befriend others who have similar tastes, or do their tastes become more similar after they become friends? In this paper, we investigate the correlation between online user trust formation and their common interests, measured by the overlap rate *ρ* and taste similarity *θ* respectively. The trust relation creation time is set as the zero timestamp. The statistical results before and after the trust formation for an online network, namely *Epinions*, show that, the overlap rate *ρ* increases greatly before the trust formation, while it would increase smoothly after the creation of the trust relation. Comparing with the empirical results, two null models are presented by shuffling the temporal behaviors of online users, which suggests that the accumulation of the common interests can result in the trust formation. Furthermore, we investigate the taste similarity *θ* of the common interests, which can reflect the users’ preference on their common interests. The empirical results show that the taste similarity *θ* is rapidly increased around the day when users trust the others. That is, the similar tastes on the common interests among users lead to the trust formation. Finally, we report that the user degree can also influence the effect of the taste similarity *θ* on user trust formation. This work may shed some light for deeply understanding the evolution mechanism of the online social systems.

## Introduction

The social networks, such as *Facebook, Twitter, Epinions* etc., enable people not only to upload, disseminate and share what they like but also to create relations with each other [1, 2]. Thus the footprints of all anticipants’ online activities can be recorded, which will provide prototypes of real networked complex systems. Therefore, both theoretical and experimental works have been carried out to investigate the human interests and social relations in online social networks [3–6]. Especially, the correlations between common interests among users and the formation of interpersonal contacts are widely studied [4, 7, 8]. For example, Mark [9] found that social relations can be helpful to diffuse the musical preference among social members. Meanwhile, using the local trust matrix techniques, the effect of initial interpersonal trust relations of users on trust formation prediction was interpreted [10]. Recently, the effects, introduced by the peer influence and the social homophily, on the relation formation have been investigated [3, 11], in which peer influence affects the students’ friendship less effective than the common interests. And people are likely to frequently connecting with the other people who have more commonalities with them [8].

Although the common interests play an important role on the formation of relations, the distinct priority between common interests and relation formation has not been well-interpreted. Many prior works investigating the correlations between common interests and the creation of social relations only focused on specific groups such as druggies or students in campus [3, 12, 13], which are more probable for them to have the similar value orientation, culture tastes and behavioral idioms etc. Thus it is easy to find the similar characteristics when we refer to their relation creations. Instead, the trust relations investigated in this paper involve millions of people who registered in a popular social networks, *Epinions*, which suggests that the website can be exposed to all kinds of persons. Additionally, the results of some survey-based network researches can be influenced by common external factors such as interviewer effects, recall limitation and other influences [14]. The online users’ behaviors are produced and recorded spontaneously regardless of the external factors. Furthermore, instead of using the interaction “events” such as e-mail or instant messaging to imply an underlying structure of relationships [7, 11], the *Epinions* data refers to explicit “trust relationships”.

**Fig 1:**
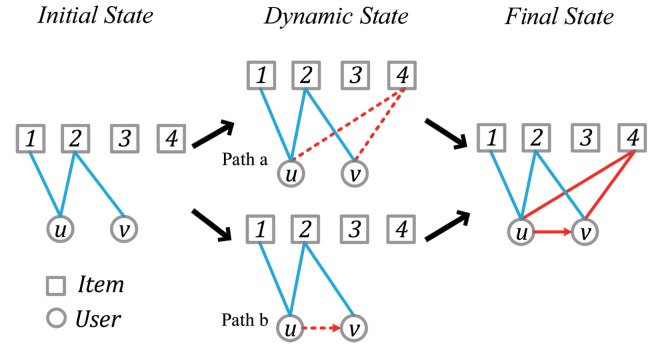
Schematic illustration of the probable coevolving process of common interests and trust relations in this paper. Suppose there are a pair of users, say user *u* and user *v*, and four reviews as the items. We assume that the items both user *u* and user *v* commented are the common interests for them. At Initial state, both user *u* and user *v* comment item 2 as well as user *u* also comments item 1. The overlap between the two users equals the ratio for the numbers of the items commented by user *v* and user *u*, i.e., $\frac{1}{2}=0.5$. At Final state, the value of overlap between the two users has increased to $\frac{2}{3}=0.67$ since both of them commented item 4. Meanwhile, user *u* has trusted user *v*. However, there are two probable ways for the two users to reach the Final state from the Initial state, say Path *a* and Path *b*. Path *a* indicates that the increment of the common interests between users can bring new trust relation; Path *b* implies that the creation of trust relation is the reason for users to have more common interests.

Generally, online systems allow users to not only befriend each other but also select movies, music and comment reviews in terms of their preferences. This kind of systems are also known as coupled social networks (*CSN*), which are of great importance for the analysis to recommender systems. In *CSN*, one’s personal preference on specific item can be affected by both his own attributes and his friends’ preference [15]. Considering the social influence and the user’s personal preference, Nie et al. [16] proposed a hybrid algorithm to provide more accurate recommendations. Actually, the evolution properties of *CSN* often involve the dynamics of a two-layer network, i.e., the layer of the user-user network where users can create social relationships and the layer of the user-item network where users can select, comment and rate on their favorite items. Therefore, in *Epinions*, by tracking the user comment behaviors and the social relation evolvements, we could explore the correlation between the trust formation and common interest changes. The evolving process of the *Epinions* user is shown in Fig 1, in which two paths are available for the user comment behaviors from the Initial State to the Final State. The path *a* shows that a pair of users both comment item 4 before the creation of trust relation, which implies that the accumulation of common interests brings a new relation. The path *b* shows that the pair of users both comment on item 4 after the creation of trust relation, which indicates that the creation of relation is the reason for the increment on common interests.

To confirm which path is the real process from the Initial state to the Final state, we investigate the dynamics of the common interest overlaps around the relation creation time for online network users. The empirical results comparing with two null models indicate that, the trust formation relies on the accumulation of common interests between two users. Meanwhile, we empirically analyze the dynamics of user taste similarity on common interests, and found that the taste similarity is another essential reason for online trust formation. Finally, we discover that the effect of the taste similarity on user trust formation can be influenced by the user degree.

## Materials

The data set used in this paper is originated from a product review web site named *Epinions* (http://www.trustlet.org/wiki/Extended_Epinions_dataset). Generally, the web users take part in four patterns of online activities: writing reviews about products, commenting reviews, expressing their trust to other users and rating reviews (1-Not helpful, 2-Somewhat helpful, 3-Helpful, 4-Very helpful, 5-Most helpful). In the *Epinions* data set, there are 415076 users who delivered 13664916 ratings on 1560182 reviews before August 12th, 2003. Meanwhile, these users have created 717620 trust relations. We regard the data before January 17th, 2001 as the basement and explore the common interest dynamics of 938 days from January 17th, 2001 to August 12th, 2003. We set January 17th, 2001 as the 1st day, January 18th, 2001 as the 2nd day and August 12th, 2003 as the 938th day and so on. In order to ensure the accuracy of the results, we only take into account the users who comment at least one hundred reviews and create at least one trust relation. In this paper, the reviews in particular are considered to be the items in the user-item network.

## Results

### Dynamics of overlap rate

The *Epinions* data set contains both the information of relationships among users and the information of users’ comments and ratings on reviews. This special structure is very helpful to analyze the dynamics of common interest overlaps. In this paper, we only focus on the difference of common interest overlaps before and after the relation creation time between each pair of users, say user *u* and user *v*. The review that both of them have commented is regarded as a unit to measure their common interests. Then, the overlap rate *ρ*_*uv*_(*t*) of user *u* and user *v* at time *t* can be denoted by

$$\begin{array}{c} \rho_{uv}\left(t\right)=\sum_{i=1}^{t}n\left(u,v,i\right)/k\left(u,t\right), \end{array}$$ (1)

where *t* ∈ {1, 2, ⋯, 938} and *n*(*u*, *v*, *t*) denotes the number of the reviews at time *t* that satisfies two requirements as follows: 1) the reviews must be commented by both user *u* and user *v*; 2) the time that user *u* commented the reviews can not be earlier than the one of user *v*. The degree *k*(*u*, *t*) is the number of the reviews that user *u* has commented before the (*t*+1)th day. Thus the average overlap rate *ρ*(*t*) for each day is equal to

$$\begin{array}{c} \rho \left(t\right)=\frac{1}{E}\sum_{u=1}^{N}\sum_{v=1}^{N}\rho_{uv}\left(t\right), \end{array}$$ (2)

where *E* is the number of the trust relations that we counted, *N* is the number of the users in the system. In order to compare the difference between the overlap rate before and after the trust formation, we define a relative time series *t*_*c*_ for each trust relation. The creation time of the trust relation is set as *t*_*c*_ = 0. The days before and after *t*_*c*_ = 0 are regarded as *t*_*c*_ = −1 and *t*_*c*_ = 1 and so on. We take the relative time *t*_*c*_ as *t*_*c*_ ∈ {−25, −24, ⋯,0, ⋯,24,25}. Then the time interval for each trust relation becomes a relative time window from -25 to 25. Correspondingly, we investigate the trust relations created during *t* = 26 and *t* = 912, which can account for 92.99% of all trust relations (see details in S1 Fig and S1 Text).

**Fig 2:**
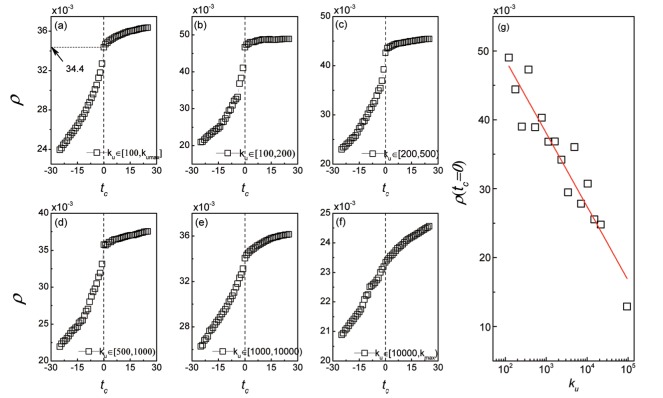
The dynamics of the overlap rate *ρ* before and after the creation of trust relations. The trust relation creation time is regarded as time *t*_*c*_ = 0. (a) The growth of overlap rate *ρ* goes increasingly rapid before time *t*_*c*_ = 0, while it will grows smoothly after the trust formation. The different growth patterns of the overlap rate *ρ* before and after time *t*_*c*_ = 0 imply that, it is the accumulation of the common interests among users results in the trust formation, and not the reverse. (b)-(f) The detailed results of the dynamics of overlap rate *ρ*, in which the users are divided by their degrees into different groups, where the users’ degrees are in ranges [100, 200), [200, 500), [500, 1000), [1000, 10000) and over 10000 respectively. The dynamics of the overlap rate exhibit similar trend in form for different user groups except the one with the user degree ≥ 10000. (g) For each pair of users, saying user *u* and user *v*, when user *u* trusts user *v*, the overlap rate *ρ* will decrease as the user degree *k*_*u*_ increases.

The dynamics of the overlap rate *ρ* before and after time *t*_*c*_ = 0 are shown in Fig 2(a). The overlap rate *ρ* continues to increase as the relative time *t*_*c*_ increases. One could find that the growth processes of the overlap rate *ρ* before and after *t*_*c*_ = 0 are explicitly different. Before *t*_*c*_ = 0, the overlap rate *ρ* grows from 0.0239 to 0.0344, while the overlap rate *ρ* only increases from 0.0344 to 0.0364 during the rest 25 days. The increasement ratio of the overlap rate *ρ* before and after *t*_*c*_ = 0 is $\frac{0.0105}{0.0020}=5.25$, which suggests that the rapid growth of common interest overlaps before the user trust formation leads to the creation of trust relation, and not the reverse. In addition, we divide the user set into groups in term of the user degree to investigate the overlap rate *ρ* more specifically, where the user degrees are set as [100, 200), [200, 500), [500, 1000), [1000, 10000) and over 10000, respectively. The detailed results are shown in Fig 2(b)–2(f), from which, one can find that, the different growth patterns of common interest overlaps before and after the trust formation are almost similar to different user groups. However, when a user trusts another user, the overlap rate *ρ* between them will decreases as their degrees increase (see Fig 2(g) and more results in S2 Fig and S2 Text). That is, the role of common interest overlaps on trust formation is relatively significant for users with small degrees.

**Fig 3:**
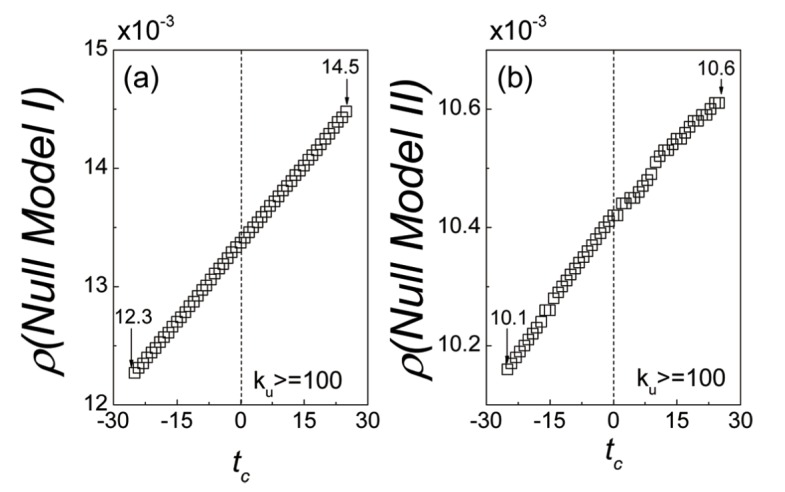
The dynamics of overlap rate *ρ* for Null models. The model I is constructed by shuffling the timestamps of the user’s comment series into random order. Thus the temporal patterns of the user comment behaviors are removed. The model II is generated by randomizing the trust relation creation time for each pair of users, so that the users’ temporal trust behaviors are totally random. (a) The variation of overlap rate *ρ* for trust relations in Null model I. (b) The variation of overlap rate *ρ* for trust relations in Null model II. In both Null models, the overlap rate *ρ* linearly increases as the time *t*_*c*_ increases. The results are totally different with that of the empirical data, which suggests that, if users perform randomized comment behaviors or trust behaviors, there will be no correlation between the trust formation and the accumulation of common interests. In other words, only in the real cases, the increasement of common interest overlaps will lead to the trust formation.

To compare with the empirical results, two null models are introduced in the following ways. Firstly, for both null models, the users and items equal that of the real data set. Secondly, the user comment behaviors on items and the trust relations for each pair of users are unchangeable. Then the model I is constructed by shuffling the timestamps of the user’s comment series into random order. Thus the temporal patterns of the user comment and rating behaviors are removed. The model II is the case in which we shuffle the trust relation creation time for each pair of users, so that the users’ temporal trust behaviors are totally random. The dynamics of overlap rate for null models are shown in Fig 3. It can be seen that, for the model I and model II, the overlap rate *ρ* both linearly increases as the time *t*_*c*_ increases, and the values of the overlap rate *ρ* grows from 0.0123 to 0.0145 and from 0.0101 to 0.0106 respectively. As a result, the growth process of overlap rate *ρ* shows no difference before and after the relation creation time in both models. That is to say, the empirical results can not be reproduced by the user’s random temporal behaviors. We can conclude from the comparisons between the null models and the empirical data that, in the real cases, the increasement of common interest overlaps lead to the trust formation.

### Dynamics of user taste similarity

From the above analysis, we know that the growth patterns of overlap rate *ρ* have big differences before and after the formation of trust relations. Especially on the day when users established the trust relation, the overlap rate *ρ* of common interests would rapidly increase. However, the above results only take into account the comment behaviors, namely the overlaps of the common interests, generated by all users. In fact, the online user tastes, measured by the rating values, also affect the user’s collective behaviors. For example, both user *u* and user *v* rate 1 to one item, their tastes are similar. However, their tastes would be totally different when they rate 1 and 5 respectively. Therefore, the taste similarity of two users on their common interests is necessary for the trust formation analysis. In this paper, we use the Pearson Correlation Coefficient (*PCC*) to measure the taste similarity for a pair of users. Thus the greater the *PCC* is, the more similar the users’ tastes are. And then, we can track the variation of the taste similarity before and after the creation of the trust relation.

In the *Epinions* data set, the ratings given by users are specific scores (1-Not helpful, 2-Somewhat helpful, 3-Helpful, 4-Very helpful, 5-Most helpful). For each trust relation, we calculate the correlation between the rating vectors of each pair of users, measured by the *PCC* index. For instance, with the trust relation, say user *u* trusts user *v*, we analyze the cumulative changes of the *PCC* for rating vectors between user *u* and user *v*. The time window ranges from *t*_*c*_ = −25 to *t*_*c*_ = 25. For a certain day *t*_*c*_, both user *u* and user *v* rated *M*_*t*_*c*__ reviews before time *t*_*c*_+1. Thus the sequences of these ratings constitute two vectors, *R*_*u*_(*t*_*c*_) = {*r*_*u*1_, *r*_*u*2_, ⋯, *r*_*uM*_*t*_*c*___} for user *u*, and *R*_*v*_(*t*_*c*_) = {*r*_*v*1_, *r*_*v*2_, ⋯, *r*_*vM*_*t*_*c*___} for user *v*. We can measure the taste similarity between the two users by calculating the *PCC* between vector *R*_*u*_ and vector *R*_*v*_. Then the taste similarity *θ*_*uv*_(*t*_*c*_) between user *u* and user *v* at time *t*_*c*_ can be described as

$$\begin{array}{c} \theta_{uv}\left(t_{c}\right)=\frac{\frac{1}{M_{t_{c}}}\sum_{i=1}^{M_{t_{c}}}\left[\left(r_{ui}-{\bar{r}}_{u}\right)\left(r_{vi}-{\bar{r}}_{v}\right)\right]-{\bar{r}}_{u}{\bar{r}}_{v}}{\sigma_{u}\sigma_{v}}, \end{array}$$ (3)

where ${\bar{r}}_{u}=\frac{1}{M_{t_{c}}}\sum_{i=1}^{M_{t_{c}}}r_{ui}$, ${\bar{r}}_{v}=\frac{1}{M_{t_{c}}}\sum_{i=1}^{M_{t_{c}}}r_{vi}$, $\sigma_{u}=\sqrt{\frac{1}{M_{t_{c}}}\sum_{i=1}^{M_{t_{c}}}{\left(r_{ui}-{\bar{r}}_{u}\right)}^{2}}$, $\sigma_{v}=\sqrt{\frac{1}{M_{t_{c}}}\sum_{i=1}^{M_{t_{c}}}{\left(r_{vi}-{\bar{r}}_{v}\right)}^{2}}$. Thus the average taste similarity *θ*(*t*_*c*_) can be described as

$$\begin{array}{c} \theta \left(t_{c}\right)=\frac{1}{E}\sum_{u=1}^{N}\sum_{v=1}^{N}\theta_{uv}\left(t_{c}\right). \end{array}$$ (4)

**Fig 4:**
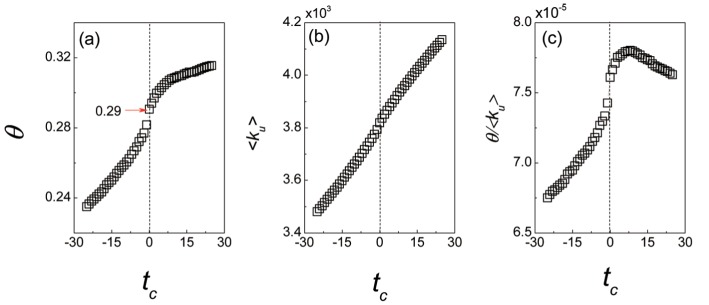
The correlation between the dynamics of the taste similarity *θ* and the trust formation. The taste similarity *θ* is quantified by the *PCC* value of the rating vectors on common interests for a pair of users. (a) The dynamics of the taste similarity *θ* as the time *t*_*c*_ increases. From *t*_*c*_ = −25 to *t*_*c*_ = 0, the taste similarity *θ* rapidly increased from 0.2350 to 0.2906. Then the taste similarity *θ* only increases by 0.0249 from *t*_*c*_ = 0 to *t*_*c*_ = 25. The comparison of the growth rate of the taste similarity *θ* before and after the time *t*_*c*_ = 0 indicates that, the users’ similar tastes lead to the creation of trust relation and not the reverse. (b) The average number of the items commented by users as the time *t*_*c*_ increases. In *Epinions*, the average number of the items commented by users, i.e., the average degree of users, ⟨*k*_*u*_⟩ linearly increases as the time *t*_*c*_ increases. (c) The dynamics of the ratio *θ*/⟨*k*_*u*_⟩ between the taste similarity and the average degree. Before time *t*_*c*_ = 0, the ratio *θ*/⟨*k*_*u*_⟩ increases rapidly, which means that, the users’ taste similarity increasingly close despite the number of items they comment increases.

**Fig 5:**
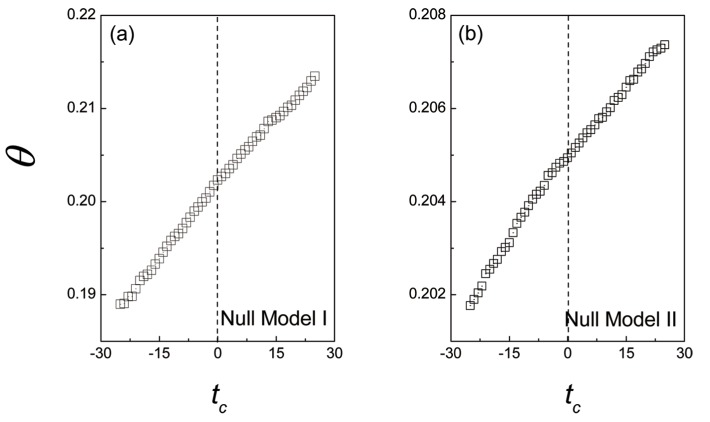
The dynamics of the taste similarity *θ* for Null models. The design of the null models have been introduced in the section *Dynamics of common interest overlaps*. (a) and (b) The dynamics of the taste similarity *θ* for Null model I and Null model II, respectively. In both Null models, the taste similarity *θ* linearly increases as the time *t*_*c*_ increases, which is totally different with the result shown by the empirical data. Therefore, the users’ real tastes can not be reflected by their random online temporal behaviors.

Fig 4(a) shows the dynamics of the taste similarity *θ* as the relative time *t*_*c*_ increases, from which, one can find that the taste similarity *θ* keeps increasing as the time *t*_*c*_ increases. However, the growth of the similarity *θ* exhibits distinct process before and after time *t*_*c*_ = 0. From *t*_*c*_ = −25 to *t*_*c*_ = 0, the taste similarity *θ* rapidly grows from 0.2350 to 0.2906, increasing by 0.0556. Then the taste similarity *θ* only increases by 0.0249 from *t*_*c*_ = 0 to *t*_*c*_ = 25. The result means that, before a user trusts another user, their tastes on the common interests increasingly close. The difference between the growth processes of taste similarity *θ* before and after time *t*_*c*_ = 0 indicates that, the users’ similar tastes can result in the trust formation. Additionally, the results of the null models (the null models have been introduced in the section *Dynamics of overlap rates*) for taste similarity *θ* are shown in Fig 5. In the null models, the taste similarity *θ* linearly grows as the time *t*_*c*_ increases. The comparison between the null models and the real data implies that, the growth of the taste similarity *θ* in empirical result can reflect the role of the temporal rating patterns, which involves users’ real tastes on their common interests, in trust formation.

However, the above result for the taste similarity may be influenced by the evolvements of the system. For example, for a pair of users, assume that the reviews both of them commented increase as the time goes, then if their tastes on the newly commented reviews are different, the taste similarity *θ* of the two users will decline. As a result, we compare the dynamics of the average user degree ⟨*k*_*u*_⟩ with that of the taste similarity *θ*. The results are shown in Fig 4(b)–4(c). It can be seen that the average degree ⟨*k*_*u*_⟩ linearly increases as the time *t*_*c*_ increases. Meanwhile, before *t*_*c*_ = 0, the ratio *θ*/⟨*k*_*u*_⟩ between the taste similarity and the average user degree also grows rapidly as the time *t*_*c*_ increases. That is to say, on the collective level, before the creation of the trust relation, not merely the average number of items commented by each user increases, but the users’ tastes on the newly selected items are increasingly similar. In other words, the growth of the taste similarity *θ* is originated from the users’ similar tastes regardless of the evolvements of the system.

All aforementioned results suggest that not only the overlap rate *ρ* but also the taste similarity *θ* affect the trust formation. In fact, both the overlap rate *ρ* and the taste similarity *θ* capture the characteristics of the common interests among users. However, the taste similarity can reflect the users’ real preference on common interests, which is more essential for the trust formation than the overlap rate that just reflects the user comment behaviors.

### Effect of user degree on trust formation

All the above findings indicate that, the role of common interests in trust formation highlights the indispensable effects of the overlap rate *ρ* and the taste similarity *θ*. As a matter of fact, the users’ online comment behaviors are also correlated with the statistical properties of the social networks [17]. Especially, individuals with different degrees in network often represent different behavior patterns [18, 19]. Therefore it is necessary to inspect the effect of the user degree on trust formation.

**Fig 6:**
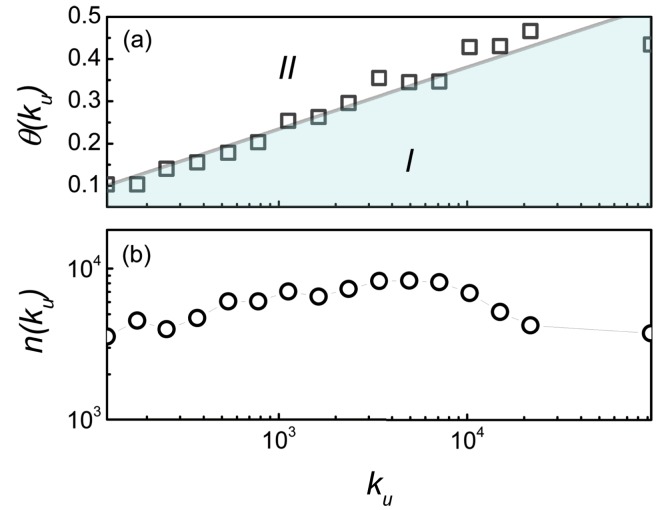
The correlation between the user degree and the taste similarity *θ* when the relations are created. The users are separated into 16 groups in terms of their degrees *k*_*u*_. The step size of the degree within each group is set as $d=\frac{1}{16}\text{lg}\frac{k_{u}^{max}}{k_{u}^{min}}$, then the degree interval of the *n* ^*th*^ group is [10^*n*_0_+*nd*^,10^*n*_0_+(*n*+1)*d*^], where *n*_0_ = 2 and *n* ∈ {0, 1, ⋯, 15}. And only the average taste similarity *θ* at the relation creation time is considered for each group. (a) The joint impact of the taste similarity *θ* and the user degree *k*_*u*_ on trust formation. At the creation time of trust relations, the taste similarity *θ* continues to increase as the user degree *k*_*u*_ increases. And the correlation between the taste similarity *θ* and the user degree *k*_*u*_ is well fitted by a straight line. Thus the users relation status can be regarded as two status, that is, the unstable status in which the users’ taste similarity are not reach the value corresponding to the degree (area I), and the trust status in which the trust relation is created between a pair of users since their taste similarity exceeds the corresponding value (area II). (b) The number of relations counted for each group.

We investigate the correlation between the user degree *k*_*u*_ and the taste similarity *θ*. In detail, we specially take into account the taste similarity *θ* for each pair of users at the relation creation time. And the users are divided into 16 groups in order to capture the average taste similarities with different user degree collectively. The result is shown in Fig 6(a). One could find that, at the relation creation time, the taste similarity *θ* keeps increasing as the user degree *k*_*u*_ increases. That is, when it refers to the trust formation, the large-degree users lay emphases on the similar tastes of common interests with his friends more than whom with small-degree. Moreover, we report that there are two relation statuses decided by the user degree *k*_*u*_ and the taste similarity *θ*. One is the unstable status, in which the taste similarity *θ* for one user does not reach the value corresponding to the user degree. That is, the value of the taste similarity locates in area I, as shown in Fig 6(a). Once the taste similarity *θ* exceeds the value corresponding to the user degree, the user would trust another user. This case can be regarded as the trust status, in which the value of taste similarity lies is in area II in Fig 6(a). Besides, Fig 6(b) shows the number of relations counted for each group, which indicates that the results are effective with enough trust relations. The above results may shed light on predicting the trust formation by both considering the user degree and the taste similarity.

## Conclusion and Discussion

The role of common interests is important for the creation of online social relations. In this paper, we empirically investigated the correlation between the users’ common interests and the online trust formation.

Firstly, we defined the overlap level of common interests among users as the overlap rate *ρ*, originated by the user comment behaviors. Thus, the dynamics of the common interests before and after the trust formation can be captured by the variation of overlap rate *ρ*. The empirical results showed that, before the creation of trust relations, the overlap rate *ρ* increased by 0.0105, and after the trust formation the overlap rate *ρ* only increased by 0.0020. Ratio of the increasements of overlap rate *ρ* before and after the trust formation is about 5.25, which indicated that, the rapid accumulation of common interests could lead to trust relations. Furthermore, two null models were presented to compare with the empirical results, in which we removed the temporal comment behaviors and the temporal trust behaviors for all users. The results in null models showed that there was no correlation between the trust formation and the common interests. From the comparisons between the null models and the empirical data, we concluded that, the role of common interests in trust formation was not the result of users’ random temporal behaviors but the consequence of users’ real online activities.

Secondly, since the online user tastes on items, generated by the rating values, could not be reflected by the overlap rate, we investigated the taste similarity *θ* of the common interests among users before and after the relation creation time. And the taste similarity *θ* for a pair of users was measured by the *PCC* of their rating vectors. The result showed that, the similarity *θ* increased by 0.0556 before the creation of trust relation, which is 2.23 times than the increasement after the trust formation. With the result, we reported that, the users’ similar tastes on their common interests were indispensable for trust formation, which could be more essential than the common interest overlaps.

Finally, we found that the user degree could also influence the effect of the taste similarity on trust formation. In brief, the accumulation of the common interests among users, captured by both of the dynamics of the overlap rate *ρ* and the taste similarity *θ*, can lead to the trust formation and not the reverse.

However, the empirical analysis may be affected by the uncontrollable external factors, for example, the current fashion trend always has important impact on users’ tastes on items [20–23], and further may influence their comments on another user. Besides, the physical limitation of human on maintain the maximum number of social relations, which is famous as the Dunbar’s number [24–26], may confine the effect of common interests on trust formation to a certain level (see details in S5 Fig, S5 Text and S1 Table). More importantly, the microscopic model has not been proposed to uncover the mechanism of the correlation between common interests and the creation of online relations. The variation trend of common interest overlap rate may fluctuate when the time interval gets longer, which means that users may create trust relations not only once in all life span. The above aspects should be paid more attention on further studies.

Despite the limitations, our results provide an empirical view for understanding the reason why people create interpersonal relations. Given that we consider human trust behaviors in an online context that are increasingly influential for the conduct of daily life [27], our results are somewhat consistent with the common notion that what we like associates with those like-minded. With the special effect of common interests on user’s trust formation, our findings may be helpful to comprehend the preference-based algorithms in recommender systems [28, 29] and the people’s collective behaviors.

## Supporting Information

S1 text The choice of the relative time windows. In the main text, the relative time window we investigated for each relation is a modified and symmetrical one around *t*_*c*_ = 0, and the time interval was set to be 51 days in total. In fact, to investigate the dynamics of users’ common interests by the method mentioned in this paper, it is ineluctable that the data on the brink of the whole 938 days would be cut off. The rest data with cutting the margin should be appropriate to interpret the properties of the relations in great majority. Thus we count the number of relations that the time set *T* = {−25, −24, ⋯, 0, ⋯, 24, 25} can account for.
We assume the time when the users first appeared in the data set as the time they entered into the system. For a relationship that user *u* trusts user *v*, the time when user *u* entered the system is denoted as *t*_0_, and the time of the trust formation *t*_*c*_ = 0 is regarded as *t*_*e*_. Then we define the *time* *gap* as *t*_*g*_ = *t*_*e*_−*t*_0_. Thus we count the frequency distribution of the trust relations with different time gap *t*_*g*_, as shown in S1 Fig. S1 Fig indicates that the bulk (92.99%) of the trust relations is characterized by time gap *t*_*g*_ ≥ 25. Therefore, with the confidence level of 92% can we conclude the results in the main text.
(DOC)

S1 Fig The frequency distribution of the number of trust relations for each time gap *t*_*g*_. For a pair of users, say user *u* and user *v*, the time gap *t*_*g*_ is denoted by the difference between the time that user *u* trusted user *v* and the time that user *u* entered into the system. Thus, only when the time gap *t*_*g*_ ≥ 25 can we calculate the overlap rate *ρ* and the taste similarity *θ* in a symmetrical time window from -25 to 25. The data with time gap *t*_*g*_ < 25, locating in the shadow, is discarded.
(PDF)

S2 TextDetailed results of the overlap rate *ρ*.We implement the same experiments of the dynamics of common interest overlap rate *ρ* with grouping the users into 16 groups in term of the user degree. Let the step size $d=\frac{1}{16}\text{lg}\frac{k_{u}^{max}}{k_{u}^{min}}$, then the degree interval of the *n*th group is [10^*n*_0_+*nd*^, 10^*n*_0_+(*n*+1)*d*^], where *n*_0_ = 2 and *n* ∈ {0, 1, ⋯, 15}. The results of dynamics of common interest overlap rate *ρ* are shown in S2(a)–S2(p) Fig for each group respectively.The results show that for the users with small-degree (see S2(a)–S2(l) Fig), the growth process of the overlap rate *ρ* exhibits similar tendency with the results shown in main text Fig 2(b)–2(e). For the users with large-degree(see S2(m)–S2(p) Fig), little difference for the growth tendency of the overlap rate *ρ* is shown before and after the creation of the trust relations. Combining the results shown in Fig 2 and in S2 Fig, we conclude that, for small-degree users, the role of the common interest overlaps on trust formation is more significant than that for the with large-degree users.(DOC)

S2 Fig The dynamics of the overlap rate *ρ* for 16 user groups. (a)-(l) The results for users with relatively small degrees, which suggest the similar patterns on the growth of the overlap rate *ρ*. That is, the growth of the overlap rate *ρ* are remarkably different before and after the trust relation creation time. (m)-(p) The results for users with degree larger than 8430, which indicate that, the difference of the growth processes of overlap rate *ρ* before and after time *t*_*c*_ = 0 tend to be less significant as the user degree increases.
(PDF)

S3 Text The supplemental results for null models. As we mentioned in the main text, the results of the null models suggest that, if the users perform randomized online behaviors, there would be no correlation between the trust formation and the accumulation of the common interests. To take insight into the conclusion more specific, we implement the experiments for null models by dividing the user into 5 groups in term of the user degree, the user degrees are set as [100, 200), [200, 500), [500, 1000), [1000, 10000) and over 10000 respectively.
The results are shown in S3(a)–S3(e) and S3(f)–S3(j) Fig for Null model I and Null model II respectively. Also, for trust relations, one can find that, the overlap rate *ρ* linearly grows as the time *t*_*c*_ increases for each user group. Furthermore, the results suggest that, for different user groups, the variation tendency of the overlap rate *ρ* is invariable whether the users create trust relation or not. From the detailed comparison between the empirical analysis and the results of null models, we can conclude that, the empirical results are robust to different users.
(DOC)

S3 Fig The dynamics of overlap rate *ρ* for different user groups in Null model I and Null model II. Users are divided by their degree into 5 groups and the user degrees are set as [100, 200), [200, 500), [500, 1000), [1000, 10000) and over 10000. (a)-(e) The detailed results of the overlap rate *ρ* for Null model I. (f)-(j) The detailed results of the overlap rate *ρ* for Null model II. In both models, the results for all the user groups show that the overlap rate *ρ* linearly increases as the time *t*_*c*_ increases, which suggest that, there is no correlation between the trust formation and the accumulation of the common interests if the users act in random temporal behaviors.
(PDF)

S4 Text In the main text, Fig 4(a) shows the strikingly different growth processes of the taste similarity *θ* before and after the creation of trust relations *t*_*c*_ = 0. We implement the experiments of the taste similarity *θ* for both null models, which is shown in S4(a)–S4(e) and S4(f)–S4(j) Fig respectively. The linear growth of similarity *θ* as the time *t*_*c*_ increases indicates that, the creation of the trust relations will be independent of the approximation of the users’ tastes if the users perform randomized temporal rating behaviors on the reviews. That is, the dynamical pattern of similarity *θ* shown in Fig 4(a) can only be the consequences of the evolvements of real tastes among users.
(DOC)

S4 Fig The detailed results of the dynamics of the taste similarity *θ* for Null models. (a)-(e) The results of the taste similarity *θ* for Null model I, and (f)-(j) The results of the taste similarity *θ* for Null model II. All the subplots show the linear correlations between the taste similarity *θ* and the relative time *t*_*c*_, rather than the remarkable patterns captured by the growth process of the taste similarity *θ* in empirical results.
(PDF)

S5 TextThe correlation between common interests and trust formation within the Dunbar’s number.Motivated by the correlation between common interests and online trust formation mentioned in the main text, we try to show the connection between the number of trust relations and common interests, which involves the Dunbar’s number [24–26]. Firstly, we preprocess the Epinions data set. Secondly, the interpretation of the connection between the common interests and the number of trust relations is addressed.The Epinions data consists of two parts. One is the user relation data set that contains the information about trust relations and the creation time for each relation. And the other one is the rating data set that contains the information of the user’s rating on the other’s reviews and the corresponding timestamps. The properties of Epinions data are organized in S1 Table. To investigate the dynamics of users’ common interests based on the relative time window *T* = {−25, −24, ⋯, 0, ⋯, 24, 25}, the data on the brink of the whole 938 days is inevitably wiped off. Therefore, for the purpose to be consistent with the data analyzed in main text, only the users who had commented at least one hundred reviews and had created at least one trust relation are taken into consideration. And the corresponding timestamps are confined from March 28th, 2003 to June 3rd, 2003.For a pair of users, say user *u* and user *v*, we count the number of reviews they both commented as their common interests. The number of trust relations that user *u* created is denoted by the out-degree $k_{u}^{out}$. Then the average number of common interests for user *u*, $w_{u}^{out}$ can be read as
$$\begin{array}{c} w_{u}^{out}=\frac{\sum_{v\in R_{u}}n\left(u,v\right)}{k_{u}^{out}}, \end{array}$$(5)
where *R*_*u*_ is the set that contains all the users who are trusted by user *u*, *n*(*u*, *v*) is the number of the reviews that user *u* and user *v* both rated. Specifically, for user *u*, the average number of common interests $w_{u}^{out}$ indicates that the average quantity level of common interests for user *u* to create one trust relation.We calculate the average number of common interests of the users in different groups. And the users are divided into eight groups according to the average number of common interest, i.e., $w_{u}^{out}$ belongs to (0, 1), [1, 10), [10, 20), [20, 50), [50, 100), [100, 200), [200, 500) and [500,+∞), respectively. Thus the average number of common interests *w* ^*out*^ for a certain group can be denoted by 
$$\begin{array}{c} w^{out}=\frac{1}{N_{i}}\sum_{u=1}^{N_{i}}w_{u}^{out}, \end{array}$$(6)
where *N*_*i*_ is the number of users to be counted in *i*th group (*i* = 1, 2, ⋯, 8). Correspondingly the average number of the trust relations *k* ^*out*^ can be defined by 
$$\begin{array}{c} k^{out}=\frac{1}{N_{i}}\sum_{u=1}^{N_{i}}k_{u}^{out}, \end{array}$$(7)
where *N*_*i*_ is the number of users to be counted in *i*th group (*i* = 1, 2, ⋯, 8). Then, the correlations between users’ out-degree and the average number of common interests are shown in S5 Fig.
S5 Fig shows that the user’s out-degree *k* ^*out*^ has been increased with the increase of the average common interests *w* ^*out*^ except for the *w* ^*out*^ lying in (0, 10). Nevertheless, the growth patterns of the out-degree *k* ^*out*^ before and after the average number of common interests *w* ^*out*^ = 310 are explicitly different. When the average number of common interests lies in [20, 310), the out-degree *k* ^*out*^ grows from 82 to 148 with the total increasement 66. However, the out-degree *k* ^*out*^ only increases by 44 from 148 to 192 when the *w* ^*out*^ lies in a much wider range [310, 869). The remarkable disparate growth patterns before and after the *k* ^*out*^ = 148 mean that the median of the Dunbar’s number 150 is of great significant. That is, once the number of relations one can maintain exceeds the median of the Dunbar’s number, the influence of common interests on forming trust relations among users is weak. Moreover, on the collective level, it cannot exceed 200 that the maximum number of trust relations one can maintain, which is identical to the conclusion of the Dunbar’s number. Thus, even the number of trust relations that one can create increases along with the average number of common interest, the limitation for users to maintain the maximum number of relations is still unchanged.(DOC)

S5 Fig The correlation between the average number of common interest *w* ^*out*^ and the user’s out-degree *k* ^*out*^. The users are grouped by their average number of common interest into eight groups, and the values of the *w* ^*out*^ lie in (0, 1), [1, 10), [10, 20), [20, 50), [50, 100), [100, 200), [200, 500) and [500,+∞), respectively. Once the number of common interest *w* ^*out*^ exceeds 10, the user’s out-degree keeps growing along with the *w* ^*out*^. Moreover, it can be seen that when the user’s out-degree is greater than 148 (approximately is the median of the Dunbar’s number 150), the growth of the user’s trust relations is much slower than before. Also, the result shows that the maximum number of trust relations one can maintain cannot collectively exceed 200, which is identical to the conclusion of the Dunbar’s number.
(PDF)

S1 Table The basic properties of the *Epinions* data set. *N* and *M* are the number of users and reviews respectively. From S1 Table, one can find that, on average, the number of trust relations that each user created is 1.77 and each one rated about 33.73 reviews. As we mentioned in the main text, we regard the data before January 17th, 2001 as the basement and only explore the users’ online behaviors in 938 days from January 17th, 2001 to August 12th, 2003.
(DOC)
