# Supplementary material for: Identifying the Role of Common Interests in Online User Trust Formation
Source: PLoS One. 2015 Jul 10;10(7):e0121105. doi: 10.1371/journal.pone.0121105 (PMC4498922; doi:10.1371/journal.pone.0121105)
Supplement: S1 text — In the main text, the relative time window we investigated for each relation is a modified and symmetrical one around t c = 0, and the time interval was set to be 51 days in total. In fact, to investigate the dynamics of users’ common interests by the method mentioned in this paper, it is ineluctable that the data on the brink of the whole 938 days would be cut off. The rest data with cutting the margin should be appropriate to interpret the properties of the relations in great majority. Thus we count the number of relations that the time set T = {−25, −24, ⋯, 0, ⋯, 24, 25} can account for. We assume the time when the users first appeared in the data set as the time they entered into the system. For a relationship that user u trusts user v, the time when user u entered the system is denoted as t 0, and the time of the trust formation t c = 0 is regarded as t e. Then we define the time gap as t g = t e−t 0. Thus we count the frequency distribution of the trust relations with different time gap t g, as shown in S1 Fig. S1 Fig indicates that the bulk (92.99%) of the trust relations is characterized by time gap t g ≥ 25. Therefore, with the confidence level of 92% can we conclude the results in the main text. (DOC) [file pone.0121105.s001.doc]

**Supporting Information S1 Text**

Lei Ji^1^, Jian-Guo Liu^1^, Lei Hou^1^, Qiang Guo^1^, Identifying the role of common interests in online user trust formation, Plos one.

1 Research Center of Complex Systems Science, University of Shanghai for Science and Technology, Shanghai, People's Republic of China

**S1 Text**

**The choice of the relative time windows.** In the main text, the relative time window we investigated for each relation is a modified and symmetrical one around
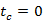
, and the time interval was set to be 51 days in total. In fact, to investigate the dynamics of users’ common interests by the method mentioned in this paper, it is ineluctable that the data on the brink of the whole 938 days would be cut off. The rest data with cutting the margin should be appropriate to interpret the properties of the relations in great majority. Thus we count the number of relations that the time set
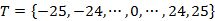
 can account for.

We assume the time when the users first appeared in the data set as the time they entered into the system. For a relationship that user *u* trusts user *v*, the time when user *u* entered the system is denoted as
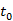
, and the time of the trust formation
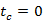
 is regarded as
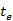
. Then we define the time gap as
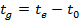
. Thus we count the frequency distribution of the trust relations with different time gap
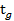
, as shown in S1 Fig. S1 Fig indicates that the bulk (92.99%) of the trust relations is characterized by time gap
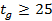
. Therefore, with the confidence level of 92% can we conclude the results in the main text.
