# Supplementary figures and images for: Identifying the Role of Common Interests in Online User Trust Formation
Source: PLoS One. 2015 Jul 10;10(7):e0121105. doi: 10.1371/journal.pone.0121105 (PMC4498922; doi:10.1371/journal.pone.0121105)

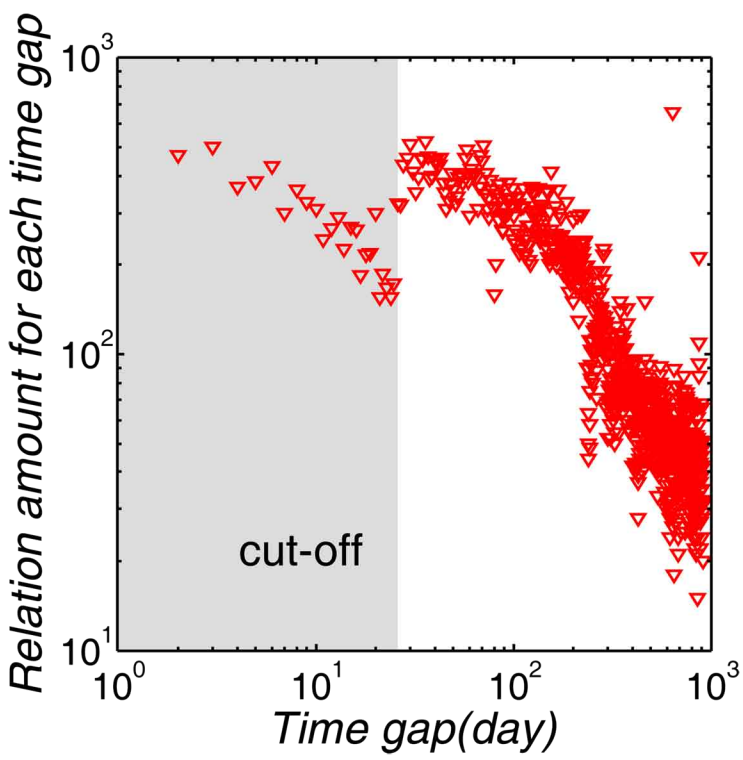

Supplement: S1 Fig — For a pair of users, say user u and user v, the time gap t g is denoted by the difference between the time that user u trusted user v and the time that user u entered into the system. Thus, only when the time gap t g ≥ 25 can we calculate the overlap rate ρ and the taste similarity θ in a symmetrical time window from -25 to 25. The data with time gap t g < 25, locating in the shadow, is discarded. (PDF) [file pone.0121105.s002.pdf]

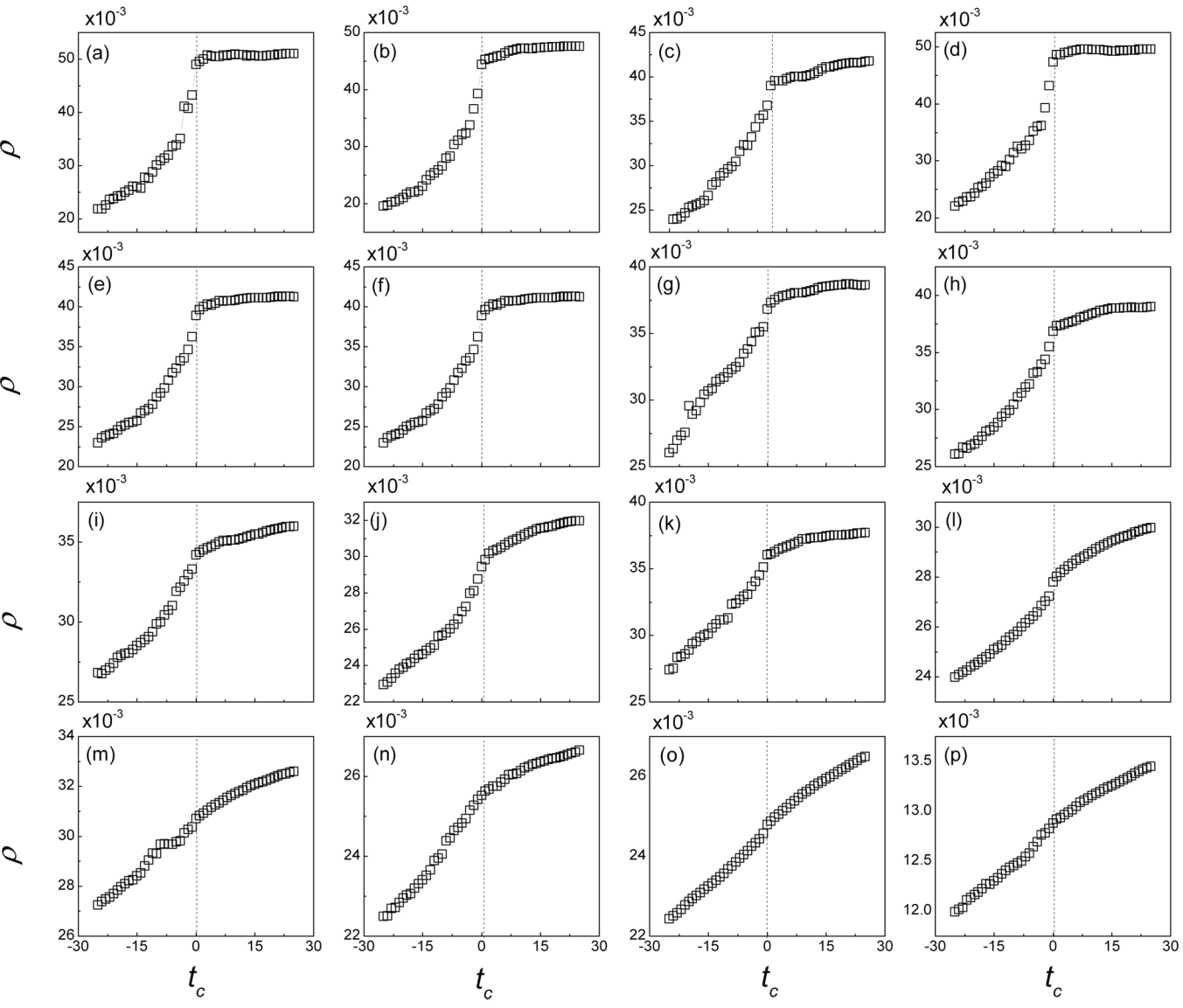

Supplement: S2 Fig — (a)-(l) The results for users with relatively small degrees, which suggest the similar patterns on the growth of the overlap rate ρ. That is, the growth of the overlap rate ρ are remarkably different before and after the trust relation creation time. (m)-(p) The results for users with degree larger than 8430, which indicate that, the difference of the growth processes of overlap rate ρ before and after time t c = 0 tend to be less significant as the user degree increases. (PDF) [file pone.0121105.s004.pdf]

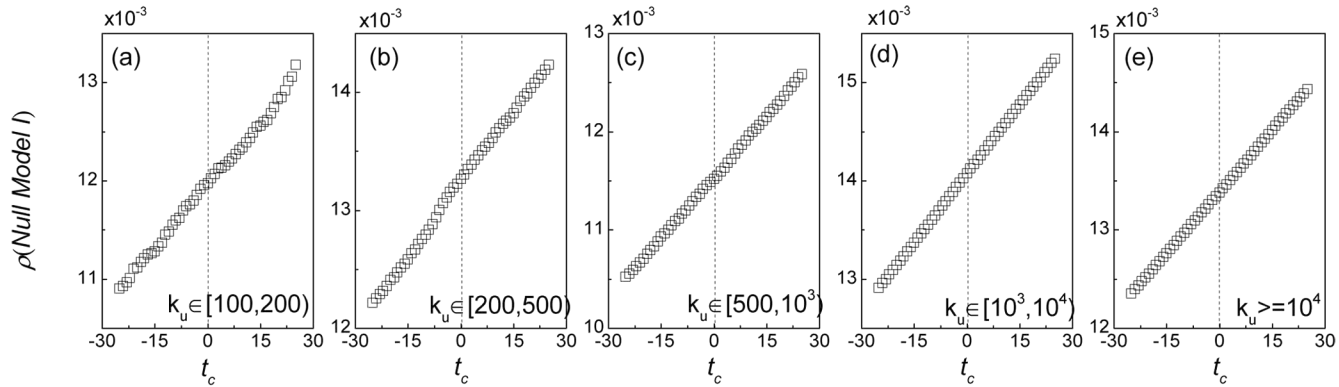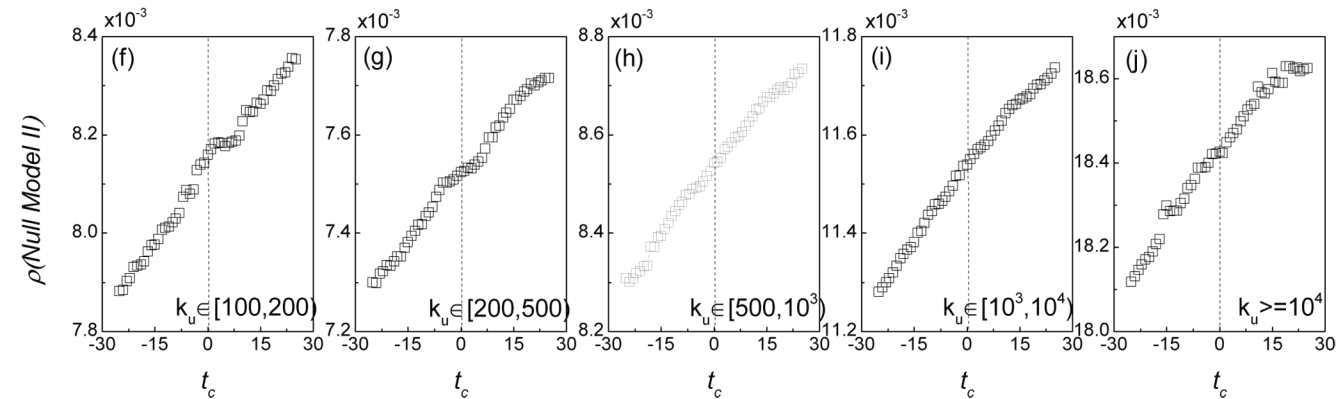

Supplement: S3 Fig — Users are divided by their degree into 5 groups and the user degrees are set as [100, 200), [200, 500), [500, 1000), [1000, 10000) and over 10000. (a)-(e) The detailed results of the overlap rate ρ for Null model I. (f)-(j) The detailed results of the overlap rate ρ for Null model II. In both models, the results for all the user groups show that the overlap rate ρ linearly increases as the time t c increases, which suggest that, there is no correlation between the trust formation and the accumulation of the common interests if the users act in random temporal behaviors. (PDF) [file pone.0121105.s006.pdf]

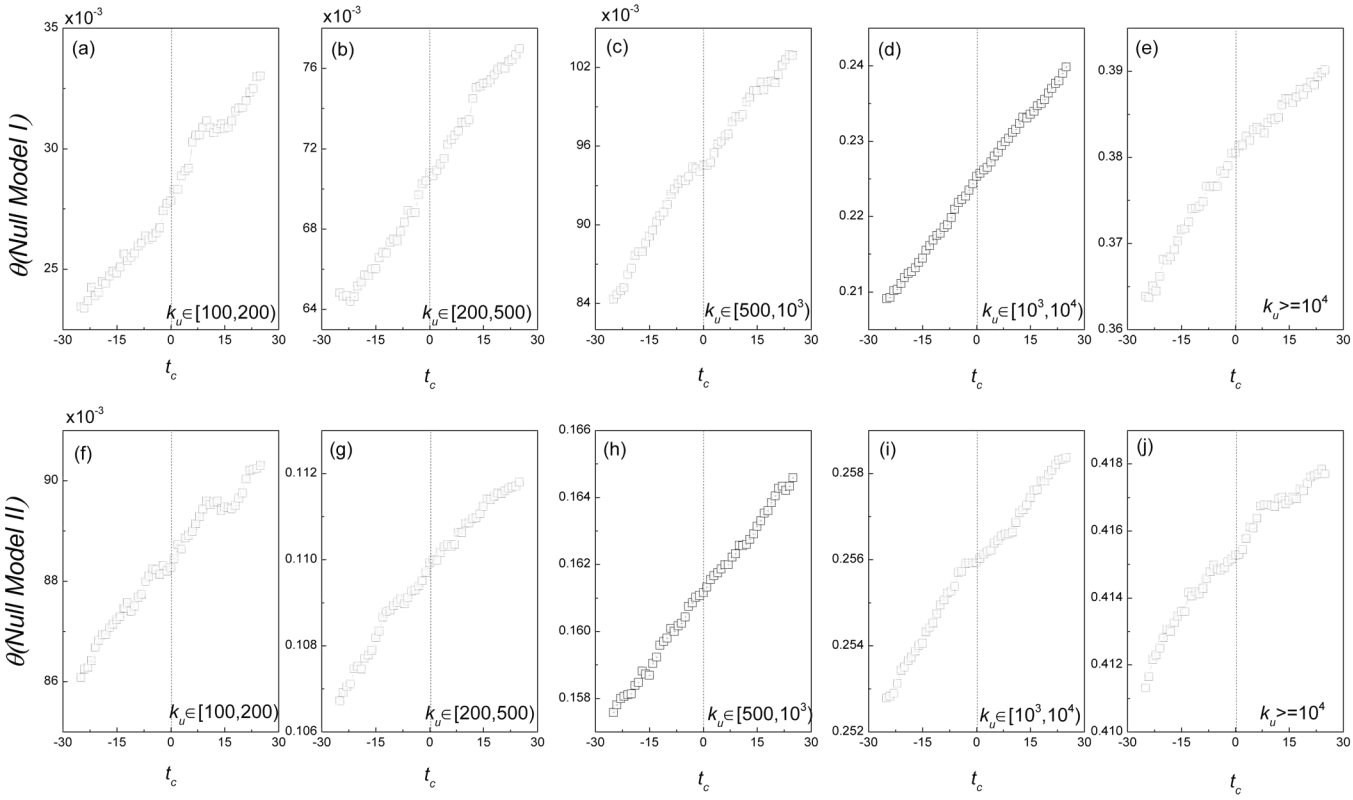

Supplement: S4 Fig — (a)-(e) The results of the taste similarity θ for Null model I, and (f)-(j) The results of the taste similarity θ for Null model II. All the subplots show the linear correlations between the taste similarity θ and the relative time t c, rather than the remarkable patterns captured by the growth process of the taste similarity θ in empirical results. (PDF) [file pone.0121105.s008.pdf]

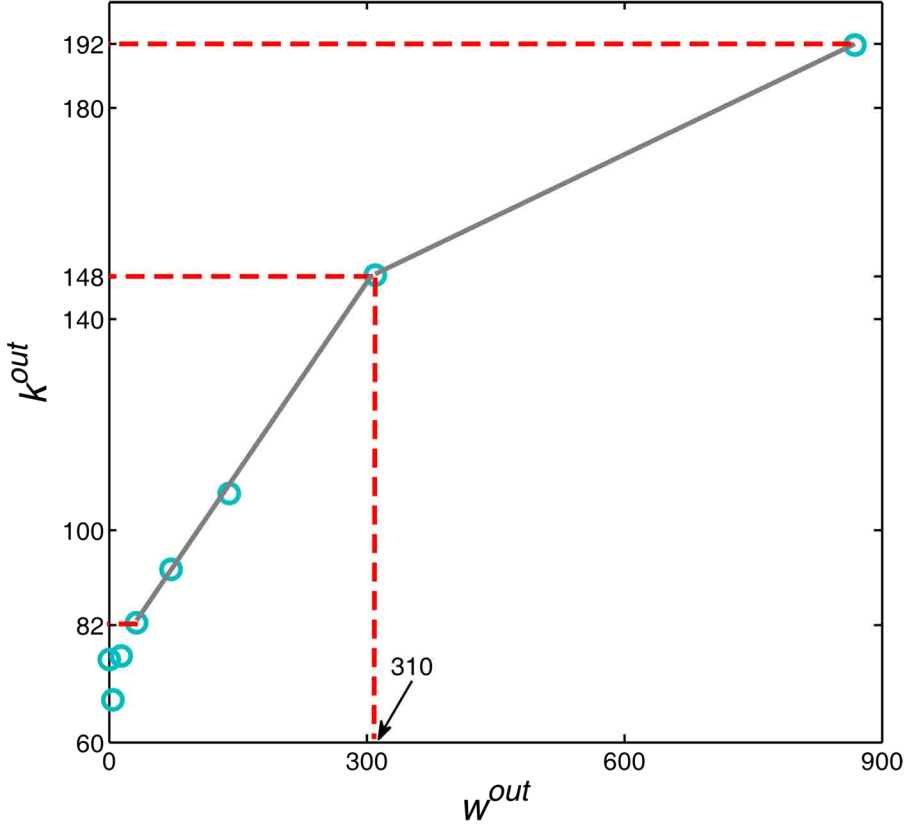

Supplement: S5 Fig — The users are grouped by their average number of common interest into eight groups, and the values of the w out lie in (0, 1), [1, 10), [10, 20), [20, 50), [50, 100), [100, 200), [200, 500) and [500,+∞), respectively. Once the number of common interest w out exceeds 10, the user’s out-degree keeps growing along with the w out. Moreover, it can be seen that when the user’s out-degree is greater than 148 (approximately is the median of the Dunbar’s number 150), the growth of the user’s trust relations is much slower than before. Also, the result shows that the maximum number of trust relations one can maintain cannot collectively exceed 200, which is identical to the conclusion of the Dunbar’s number. (PDF) [file pone.0121105.s010.pdf]
