# Supplementary material for: Identifying the Role of Common Interests in Online User Trust Formation
Source: PLoS One. 2015 Jul 10;10(7):e0121105. doi: 10.1371/journal.pone.0121105 (PMC4498922; doi:10.1371/journal.pone.0121105)
Supplement: S2 Text — We implement the same experiments of the dynamics of common interest overlap rate ρ with grouping the users into 16 groups in term of the user degree. Let the step size d=116lgkumaxkumin, then the degree interval of the nth group is [10n0+nd, 10n0+(n+1)d], where n 0 = 2 and n ∈ {0, 1, ⋯, 15}. The results of dynamics of common interest overlap rate ρ are shown in S2(a)–S2(p) Fig for each group respectively. The results show that for the users with small-degree (see S2(a)–S2(l) Fig), the growth process of the overlap rate ρ exhibits similar tendency with the results shown in main text Fig 2(b)–2(e). For the users with large-degree(see S2(m)–S2(p) Fig), little difference for the growth tendency of the overlap rate ρ is shown before and after the creation of the trust relations. Combining the results shown in Fig 2 and in S2 Fig, we conclude that, for small-degree users, the role of the common interest overlaps on trust formation is more significant than that for the with large-degree users. (DOC) [file pone.0121105.s003.doc]

**Supporting Information S2 Text**

Lei Ji^1^, Jian-Guo Liu^1^, Lei Hou^1^, Qiang Guo^1^, Identifying the role of common interests in online user trust formation, Plos one.

1 Research Center of Complex Systems Science, University of Shanghai for Science and Technology, Shanghai, People's Republic of China

**S2 Text**

**Detailed results of the overlap rate**
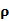
**.** We implement the same experiments of the dynamics of common interest overlap rate
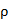
 with grouping the users into 16 groups in term of the user degree. Let the step size
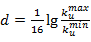
, then the degree interval of the *n*th group is
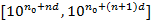
, where
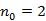
 and
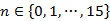
. The results of dynamics of common interest overlap rate
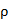
 are shown in S2 Fig (a)-(p) for each group respectively.

The results show that for the users with small-degree (see S2 Fig (a)-(l)), the growth process of the overlap rate
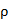
 exhibits similar tendency with the results shown in main text Fig. 2(b)-(e). For the users with large-degree (see S2 Fig (m)-(p)), little difference for the growth tendency of the overlap rate
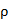
 is shown before and after the creation of the trust relations. Combining the results shown in Fig. 2 and in S2 Fig, we conclude that, for small-degree users, the role of the common interest overlaps on trust formation is more significant than that for the with large-degree users.
