# Supplementary material for: Identifying the Role of Common Interests in Online User Trust Formation
Source: PLoS One. 2015 Jul 10;10(7):e0121105. doi: 10.1371/journal.pone.0121105 (PMC4498922; doi:10.1371/journal.pone.0121105)
Supplement: S3 Text — As we mentioned in the main text, the results of the null models suggest that, if the users perform randomized online behaviors, there would be no correlation between the trust formation and the accumulation of the common interests. To take insight into the conclusion more specific, we implement the experiments for null models by dividing the user into 5 groups in term of the user degree, the user degrees are set as [100, 200), [200, 500), [500, 1000), [1000, 10000) and over 10000 respectively. The results are shown in S3(a)–S3(e) and S3(f)–S3(j) Fig for Null model I and Null model II respectively. Also, for trust relations, one can find that, the overlap rate ρ linearly grows as the time t c increases for each user group. Furthermore, the results suggest that, for different user groups, the variation tendency of the overlap rate ρ is invariable whether the users create trust relation or not. From the detailed comparison between the empirical analysis and the results of null models, we can conclude that, the empirical results are robust to different users. (DOC) [file pone.0121105.s005.doc]

**Supporting Information S3 Text**

Lei Ji^1^, Jian-Guo Liu^1^, Lei Hou^1^, Qiang Guo^1^, Identifying the role of common interests in online user trust formation, Plos one.

1 Research Center of Complex Systems Science, University of Shanghai for Science and Technology, Shanghai, People's Republic of China

**S3 Text**

**The supplemental results for null models.** As we mentioned in the main text, the results of the null models suggest that, if the users perform randomized online behaviors, there would be no correlation between the trust formation and the accumulation of the common interests. To take insight into the conclusion more specific, we implement the experiments for null models by dividing the user into 5 groups in term of the user degree, the user degrees are set as [100, 200), [200, 500), [500, 1000), [1000, 10000) and over 10000 respectively.

The results are shown in S3 Fig (a)-(e) and (f)-(j) for Null model I and Null model II respectively. Also, for trust relations, one can find that, the overlap rate
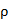
 linearly grows as the time
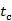
 increases for each user group. Furthermore, the results suggest that, for different user groups, the variation tendency of the overlap rate
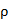
 is invariable whether the users create trust relation or not. From the detailed comparison between the empirical analysis and the results of null models, we can conclude that, the empirical results are robust to different users.
