# Supplementary material for: Identifying the Role of Common Interests in Online User Trust Formation
Source: PLoS One. 2015 Jul 10;10(7):e0121105. doi: 10.1371/journal.pone.0121105 (PMC4498922; doi:10.1371/journal.pone.0121105)
Supplement: S4 Text — We implement the experiments of the taste similarity θ for both null models, which is shown in S4(a)–S4(e) and S4(f)–S4(j) Fig respectively. The linear growth of similarity θ as the time t c increases indicates that, the creation of the trust relations will be independent of the approximation of the users’ tastes if the users perform randomized temporal rating behaviors on the reviews. That is, the dynamical pattern of similarity θ shown in Fig 4(a) can only be the consequences of the evolvements of real tastes among users. (DOC) [file pone.0121105.s007.doc]

**Supporting Information S4 Text**

Lei Ji^1^, Jian-Guo Liu^1^, Lei Hou^1^, Qiang Guo^1^, Identifying the role of common interests in online user trust formation, Plos one.

1 Research Center of Complex Systems Science, University of Shanghai for Science and Technology, Shanghai, People's Republic of China

**S4 Text**

**The dynamics of the taste similarity**
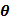
 **for Null model I and Null model II .** In the main text, figure 4(a) shows the strikingly different growth processes of the taste similarity
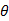
 before and after the creation of trust relations
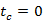
. We implement the experiments of the taste similarity
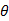
 for both null models, which is shown in S4 Fig (a)-(e) and (f)-(j) respectively. The linear growth of the taste similarity
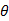
 as the time
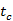
 increases indicates that, the creation of the trust relations will be independent of the approximation of the users’ tastes if the users perform randomized temporal rating behaviors on the reviews. That is, the dynamical pattern of the taste similarity
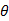
 shown in Fig. 4(a) can only be the consequences of the evolvements of real tastes among users.
