# Supplementary material for: Identifying the Role of Common Interests in Online User Trust Formation
Source: PLoS One. 2015 Jul 10;10(7):e0121105. doi: 10.1371/journal.pone.0121105 (PMC4498922; doi:10.1371/journal.pone.0121105)
Supplement: S5 Text — Motivated by the correlation between common interests and online trust formation mentioned in the main text, we try to show the connection between the number of trust relations and common interests, which involves the Dunbar’s number [24–26]. Firstly, we preprocess the Epinions data set. Secondly, the interpretation of the connection between the common interests and the number of trust relations is addressed. The Epinions data consists of two parts. One is the user relation data set that contains the information about trust relations and the creation time for each relation. And the other one is the rating data set that contains the information of the user’s rating on the other’s reviews and the corresponding timestamps. The properties of Epinions data are organized in S1 Table. To investigate the dynamics of users’ common interests based on the relative time window T = {−25, −24, ⋯, 0, ⋯, 24, 25}, the data on the brink of the whole 938 days is inevitably wiped off. Therefore, for the purpose to be consistent with the data analyzed in main text, only the users who had commented at least one hundred reviews and had created at least one trust relation are taken into consideration. And the corresponding timestamps are confined from March 28th, 2003 to June 3rd, 2003. For a pair of users, say user u and user v, we count the number of reviews they both commented as their common interests. The number of trust relations that user u created is denoted by the out-degree kuout. Then the average number of common interests for user u, wuout can be read as wuout=∑v∈Run(u,v)kuout,(5) where R u is the set that contains all the users who are trusted by user u, n(u, v) is the number of the reviews that user u and user v both rated. Specifically, for user u, the average number of common interests wuout indicates that the average quantity level of common interests for user u to create one trust relation. We calculate the average number of common interests of the users in different grou [file pone.0121105.s009.doc]

**Supporting Information S5 Text**

Lei Ji^1^, Jian-Guo Liu^1^, Lei Hou^1^, Qiang Guo^1^, Identifying the role of common interests in online user trust formation, Plos one.

1 Research Center of Complex Systems Science, University of Shanghai for Science and Technology, Shanghai, People's Republic of China

**S5 Text**

**The correlation between common interests and trust formation within the Dunbar’s number.** Motivated by the correlation between common interests and online trust formation mentioned in the main text, we try to show the connection between the number of trust relations and common interests, which involves the Dunbar’s number. Firstly, we preprocess the Epinions data set. Secondly, the interpretation of the connection between the common interests and the number of trust relations is addressed.

The Epinions data consists of two parts. One is the user relation data set that contains the information about trust relations and the creation time for each relation. And the other one is the rating data set that contains the information of the user's rating on the other's reviews and the corresponding timestamps. The properties of Epinions data are organized in S1 Table. To investigate the dynamics of users’ common interests based on the relative time window
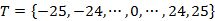
, the data on the brink of the whole 938 days is inevitably wiped off. Therefore, for the purpose to be consistent with the data analyzed in main text, only the users who had commented at least one hundred reviews and had created at least one trust relation are taken into consideration. And the corresponding timestamps are confined from March 28th, 2003 to June 3rd, 2003.

For a pair of users, say user *u* and user *v*, we count the number of reviews they both commented as their common interests. The number of trust relations that user *u* created is denoted by the out-degree
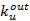
. Then the average number of common interests for user *u*,
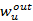
 can be read as


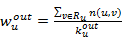
, (1)

where
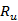
 is the set that contains all the users who are trusted by user *u*,
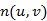
 is the number of the reviews that user *u* and user *v* both rated. Specifically, for user *u*, the average number of common interests
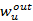
 indicates that the average quantity level of common interests for user u to create one trust relation.

We calculate the average number of common interests of the users in different groups. And the users are divided into eight groups according to the average number of common interest, i.e.,
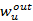
 belongs to (0, 1), [1, 10), [10, 20), [20, 50), [50, 100), [100, 200), [200, 500) and [500, +
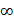
), respectively. Thus the average number of common interests
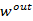
 for a certain group can be denoted by


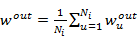
, (2)

where
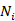
 is the number of users to be counted in *i*th group (
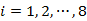
). Correspondingly the average number of the trust relations
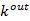
 can be defined by


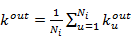
. (3)

Then, the correlations between users’ out-degree and the average number of common interests are shown in S5 Fig.

S5 Fig shows that the user’s out-degree
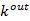
 has been increased with the increase of the average common interests
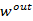
 except for the
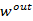
 lying in (0, 10). Nevertheless, the growth patterns of the out-degree
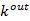
 before and after the average number of common interests
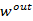
=310 are explicitly different. When the average number of common interests lies in [20, 310), the out-degree
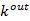
 grows from 82 to 148 with the total increment 66. However, the out-degree
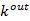
 only increases by 44 from 148 to 192 when the
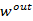
 lies in a much wider range [310, 869). The remarkable disparate growth patterns before and after the
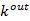
 = 148 mean that the median of the Dunbar’s number 150 is of great significant. That is, once the number of relations one can maintain exceeds the median of the Dunbar’s number, the influence of common interests on forming trust relations among users is weak. Moreover, on the collective level, it cannot exceed 200 that the maximum number of trust relations one can maintain, which is identical to the conclusion of the Dunbar’s number. Thus, even the number of trust relations that one can create increases along with the average number of common interest, the limitation for users to maintain the maximum number of relations is still unchanged.
