# Supplementary material for: Identifying the Role of Common Interests in Online User Trust Formation
Source: PLoS One. 2015 Jul 10;10(7):e0121105. doi: 10.1371/journal.pone.0121105 (PMC4498922; doi:10.1371/journal.pone.0121105)
Supplement: S1 Table — N and M are the number of users and reviews respectively. From S1 Table, one can find that, on average, the number of trust relations that each user created is 1.77 and each one rated about 33.73 reviews. As we mentioned in the main text, we regard the data before January 17th, 2001 as the basement and only explore the users’ online behaviors in 938 days from January 17th, 2001 to August 12th, 2003. (DOC) [file pone.0121105.s011.doc]

**Supporting Information S1 Table**

Lei Ji^1^, Jian-Guo Liu^1^, Lei Hou^1^, Qiang Guo^1^, Identifying the role of common interests in online user trust formation, Plos one.

1 Research Center of Complex Systems Science, University of Shanghai for Science and Technology, Shanghai, People's Republic of China

**S1 Table**

**The basic properties of the *Epinions* data set.** N and M are the number of users and reviews respectively. From table 1, one can find that, on average, the number of trust relations that each user created is 1.77 and each one rated about 33.73 reviews. As we mentioned in the main text, we regard the data before January 17th, 2001 as the basement and only explore the users' online behaviors in 938 days from January 17th, 2001 to August 12th, 2003.

| Data sets | *N* | *M* | *Links* | <k> | Time Range |
| --- | --- | --- | --- | --- | --- |
| User relation | 405176 | - | 717620 | 1.77 | Before Aug. 12, 2003 |
| Rating | 405176 | 1560182 | 13664916 | 33.73 | Before Aug. 12, 2003 |
